# Autism Data Classification Using AI Algorithms with Rules: Focused Review

**DOI:** 10.3390/bioengineering12020160

**Published:** 2025-02-07

**Authors:** Abdulhamid Alsbakhi, Fadi Thabtah, Joan Lu

**Affiliations:** 1 School of Computing and Engineering, University of Huddersfield, Huddersfield HD1 3DH, UK; j.lu@hud.ac.uk; 2 Abu Dhabi School of Management, Abu Dhabi P.O. Box 6844, United Arab Emirates; f.fayez@adsm.ac.ae

**Keywords:** ASD, classification, behavioural data, interpretable classifiers, machine learning, medical diagnosis

## Abstract

Autism Spectrum Disorder (ASD) presents challenges in early screening due to its varied nature and sophisticated early signs. From a machine-learning (ML) perspective, the primary challenges include the need for large, diverse datasets, managing the variability in ASD symptoms, providing easy-to-understand models, and ensuring ASD predictive models that can be employed across different populations. Interpretable or explainable classification algorithms, like rule-based or decision tree, play a crucial role in dealing with some of these issues by offering classification models that can be exploited by clinicians. These models offer transparency in decision-making, allowing clinicians to understand reasons behind diagnostic decisions, which is critical for trust and adoption in medical settings. In addition, interpretable classification algorithms facilitate the identification of important behavioural features and patterns associated with ASD, enabling more accurate and explainable diagnoses. However, there is a scarcity of review papers focusing on interpretable classifiers for ASD detection from a behavioural perspective. Thereby this research aimed to conduct a recent review on rule-based classification research works in order to provide added value by consolidating current research, identifying gaps, and guiding future studies. Our research would enhance the understanding of these techniques, based on data used to generate models and obtain performance by trying to highlight early detection and intervention ways for ASD. Integrating advanced AI methods like deep learning with rule-based classifiers can improve model interpretability, exploration, and accuracy in ASD-detection applications. While this hybrid approach has feature selection relevant features that can be detected in an efficient manner, rule-based classifiers can provide clinicians with transparent explanations for model decisions. This hybrid approach is critical in clinical applications like ASD, where model content is as crucial as achieving high classification accuracy.

## 1. Introduction

ASD is a disorder that influences communication, behaviour, and social interaction. Symptoms of ASD typically appear early and can vary in the level of severity and manifestation [1]. Individuals on the ASD spectrum may have difficulties in communication, repetitive behaviours, and a restricted range of interests and activities. The cause of ASD is not known, but some research works show that it may involve genetic and environmental factors. It is confirmed that early behavioural interventions can result in improvements for children on the ASD spectrum in terms of in social communication and adaptive behaviour. For example, a study by Dawson et al. [2] showed that early intervention had an average increase of 17.6 IQ points for children compared to a control group. According to the Centers for Disease Control and Prevention [3], the average age of diagnosis for ASD in the United States is around 4 years old, though early signs can be detected as early as 18 months. In the United States, approximately 1 in 54 children are diagnosed with ASD [3] thereby early screening of ASD is critical in improving developmental outcomes for these children.

Screening for ASD is critical for timely intervention, as research shows that early therapeutic strategies can improve social, communication, and cognitive outcomes in children with ASD [4]. Delayed diagnosis, often caused by limited access to specialists or subjective evaluation methods, can lead to missed intervention during developmental stages. Tools powered by ML have the potential to address these challenges by enabling accurate and objective early diagnosis for ASD. ML models, such as rule-based classifiers and deep learning, can analyze large datasets to detect early ASD, traits that may not be immediately shown to clinicians. By integrating ML algorithms into screening tools, such systems can provide real-time analysis and diagnostic recommendations. Additionally, combining ML with rule-based classifiers improves interpretability, ensuring that clinicians understand how the tools make decisions [5].

ML is a subset of artificial intelligence (AI) technology that involves the development of smart algorithms to perform specific tasks after constructing models from historical data. ML is used frequently in health-related applications, e.g., ASD classification [6,7], class imbalance in ASD data [8,9], dementia diagnosis [10]. The developed models by ML techniques can be explainable in nature and comprise rules such as those developed by rule-based algorithms [11]. These ML models’ focus is creating patterns easily understood by humans in the format of “if-then” rules, which allows for transparency and ease of interpretation. For example, a decision tree might analyze a child’s behavioural patterns and provide a clear, step-by-step rationale for diagnosing ASD based on specific criteria observed in the data [12]. A study by [13] demonstrated that Covering algorithms can generate models that could improve the clinicians’ understanding of features used for ASD assessment besides being accurate in screening ASD by analyzing large datasets and identifying key behavioural indicators. The interpretable nature of these models ensures that clinicians can understand and trust the AI’s recommendations, leading to robust and more reliable recommended diagnoses.

There are a few reviews that considered rule-based classification in ASD research. For instance, Chen et al. [5] contrasted different ML techniques such as support vector machines (SVMs), decision trees, and neural networks for their ability to identify behavioural and genetic markers. The authors used different criteria to compare ML techniques such as accuracy, efficiency, interpretability, and others. Rahman et al. [14] compared several feature selection and classification techniques used in ASD detection, focusing on their accuracy, robustness, and interpretability. Kavitha et al. [15] reviewed decision trees, SVMs, and deep learning for ASD classification using criteria including accuracy, computational efficiency, and scalability.

Most of the current review papers on ML algorithms of ASD classification including the above ones focused on measurements related to algorithms used in phases like features selection, classification, and evaluation. Examples of measurements used by most of the current works are performance (predictive accuracy), computational efficiency (training time in ms), robustness, scalability, and classification model outcome (the form of the output presenting to the user). Limited attempts have been carried out toward reviewing explainable classification techniques such as rule induction and decision tree in ASD diagnosis. To fill this gap, the aim of this work was to review recent data-driven studies on ASD classification that primarily utilized interpretable classification algorithms including hybrid AI algorithms with rules. The reason for choosing interpretable classification algorithms are the following [13,16]:

- Transparency: rules are explicit and understandable, allowing clinicians to comprehend the reasoning behind the classification decisions.
- Simplicity: rule-based systems are straightforward to implement, making them accessible and practical in clinical settings.
- Diagnostic Insight: these algorithms highlight key behavioural features and their interactions contributing to ASD classification, aiding in accurate diagnosis and intervention planning.
- Predictive Accuracy: rule-based classifiers showed models with good classification accuracy in detecting ASD.

**Figure 1:**
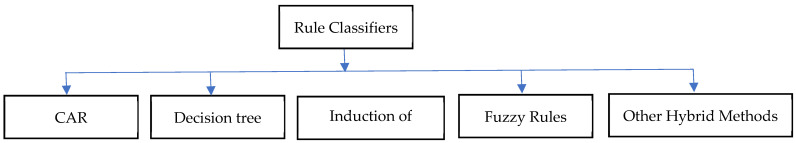
Rule-based classification-model taxonomy.

To conduct the review, we used different academic digital libraries like IEEE, ACM, World Scientific, Springer, MDPI, and others. We only considered research papers that focus on ASD classification using ML techniques that are experimental with clear data-driven methodologies. All theoretical papers have been excluded from this work. Figure 1 shows the taxonomy of classification algorithms considered in this review paper, including class association rule (CAR), decision tree, induction of rules, fuzzy rules, and other hybrid rule approaches.

The paper is organized as follows: Section 2 defines the ASD classification problem from a data-science perspective and show its main steps. Section 3 reviews rule-based classification algorithms on ASD detection, and Section 4 contains a discussion on data and methods used by the algorithms, among others. Lastly, the conclusion is provided in Section 5.

## 2. ASD Screening Problem and Steps

Classification of ASD using historical data (cases and controls) can be defined from a data-science perspective [6] as developing a predictive model to distinguish between individuals with ASD and those without using supervised learning (classification algorithm), where the dataset D = {($x_{i},y_{i}$)} i^n^ = 1 consists of feature vectors $x_{i}$ (e.g., behavioural traits and genetic features) and corresponding class labels $y_{i}$ (1 for ASD, 0 for non-ASD). Mathematically, the goal is to find a function f:X→{0,1} that minimizes the prediction error on unseen data (test data subjects).

**Figure 2:**
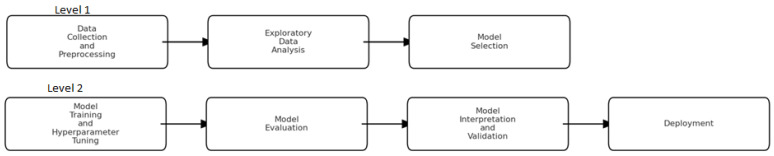
Steps needed to develop an ASD classification system.

Building a classification model for ASD diagnosis involves several steps, from data collection to model evaluation to finally deployment. Below details the steps needed based on academic work, e.g., Duda et al. [6], as shown in Figure 2. Initially, users collect data and perform preprocessing if needed. Data are collected from reliable sources such as medical records, ASD diagnostic tools (e.g., Autism Diagnostic Observation Schedule—ADOS [17], and publicly available datasets. If the data contains missing information or requires normalization or discretization, etc., the user can preprocess it in a form acceptable for the learning algorithm. Once data are prepared, then expletory analysis can be implemented to further understand the data characteristics and statistics using visualization techniques and descriptive analytic methods. Measurements like mean, median, mode, standard deviation, etc., can be utilized to understand the central tendency and variability. More essentially, this step may also involve a features assessment if needed.

Once exploratory analysis is performed, then users such as clinicians can select the appropriate classification algorithm to develop ASD models. In this step, algorithm testing methods (cross validation, etc.) and performance measurements (predictive accuracy, recall, precision, etc.) should be identified. The classification algorithms generate models that get evaluated using the chosen performance measurements. If users are happy with the performance, then these models can be employed as an integrated component inside an ASD diagnostic tool and put into operation in clinical settings.

## 3. Literature Review

### 3.1. Rule Induction Studies

The induction of rule is a classification approach that often generates models with simple-to-comprehend rules [18]. Induction of rule algorithms begin by generating initial rules to cover instances of the target class given that the input data has two or more class labels. They iteratively grow rules by adding conditions that maximize certain rule’s measurements such as rule’s expected accuracy, followed by cutting down conditions (attributes values) that do not significantly reduce error rates, thus preventing any possible overfitting.

The rule-pruning process simplifies the rules, balancing between the rule’s errors and its complexity. For example, Repeated Incremental Pruning to Produce Error Reduction (RIPPER) [19] optimizes induced rules by revisiting and refining them to reduce errors incrementally. Once the rules for one class are generated, the induction of rule algorithms proceed to the next class, excluding instances already covered by previous rules, and continues this process until all classes in the input dataset are evaluated. The iterative approach of rule growing, pruning, and optimization such as the one followed by RIPPER algorithm and its successors ensures both accuracy and efficiency, making it particularly suitable for large datasets with multiple classes.

Rule induction algorithms for ASD classification offer explanations, enabling clinicians to understand the decision-making process. These algorithms according to Duda et al. [6] and Thabtah & Peebles [13] provide clear, human-readable rules, facilitate transparent ASD diagnosis, and allow easy validation in clinical settings. Additionally, these algorithms handle noisy data effectively and can be tailored to accommodate different diagnostic criteria. Below are some recent research works on the use of rules in autism-diagnosis research.

A classifier with rules study to improve autism-detection accuracy in pediatric populations was conducted by Karre & Ramadevi [20]. The challenge addressed was accurately identifying autism in children, considering its diverse manifestations. The study utilized algorithms like RIPPER and C4.5 to develop the detection model. The dataset used clinical and behavioural features of children diagnosed with autism, and the empirical results showed that the RIPPER classifier achieved an 85% accuracy rate in detecting autism in children. A more thorough study, developed a rule-based algorithm and compared it to several other different rule-based classifiers on an ASD screening dataset, was conducted by [13]. The authors evaluated the proposed algorithm on adult and adolescent datasets, and the derived models achieved an accuracy of 95.52% on the adult dataset and 97.23% on the adolescent dataset. These results outperformed models generated by other rule-based and non-rule-based models like decision trees, Naïve Bayes, Logistic Regression, k-Nearest Neighbours (kNN), and RIPPERS, among others. The comparison revealed the effectiveness and interpretability of the rule induction approach in the context of ASD screening.

A rule-based expert system for ASD classification that used a dataset consisting of behavioural and demographic information from standardized autism screening questionnaires was explored by Adamu et al. [21]. The methodology followed by the authors involved developing a prototype expert system that employs rule-based algorithms to analyze the input data and generate diagnostic recommendations. The system’s rules are derived from established diagnostic criteria and expert knowledge. The prototype was evaluated using a set of test cases, achieving an accuracy of 92%, precision of 91%, recall of 93%, and an F1-score of 92%.

A comprehensive ASD dataset to assess several classifiers, including decision trees, Random Forest, SVMs, and kNN, in order to develop an ensemble-based recommender system, was utilized by Shinde & Patil [22]. The system combined the strengths of various classifiers to improve diagnostic accuracy. The performance of the multi-classifier system was evaluated using metrics such as accuracy, precision, recall, and F1-score. The results demonstrated a high performance, with the ensemble model achieving an accuracy of 96%, precision of 95%, recall of 97%, and an F1-score of 96%.

Cognitive challenges faced by individuals with autism, specifically in learning and generalizing rules based on repetition, was investigated by Bettoni et al. [23]. The study addressed the issue of how individuals with autism perceive and process repetitive patterns compared to neurotypical individuals. The researchers employed experimental tasks where participants, both autistic and neurotypical, were required to learn and apply rules based on repetitive sequences, using structured tests and observational analysis. The study involved individuals diagnosed with ASD and a control group of neurotypical participants, with behavioural responses and accuracy rates recorded and analyzed. The findings indicated that individuals with autism showed significant differences in learning and generalizing repetition-based rules, with accuracy rates for autistic participants being, on average, 20% lower than those of the neurotypical group.

### 3.2. Decision Trees Studies

Decision trees are a classification that usually grow a tree structure from historical data and then convert it into a set of rules to ease the simplicity of the models [24]. As a supervised learning algorithm, decision trees work by recursively partitioning the dataset into subsets based on the value of input features and a data split measurement (Gini Index [25] Shannon Entropy [26], etc.), constructing a tree-like model of decisions and their possible decisions. The structure of a decision tree comprises nodes representing attributes, branches denoting the attribute values, and leaf nodes indicating class labels or continuous values. This hierarchical arrangement enables decision tree algorithms to capture complex decision-making processes in a comprehensible manner, making them valuable for both predictions and knowledge discovery [27]. Key advantages of decision trees include their ability to handle both numerical and categorical data, manage missing values, and model non-linear relationships. Their transparent nature facilitates simple interpretation, critical for health applications where understanding the rationale behind clinical diagnosis is essential.

A study that explored distinguishing between different subtypes ASD by employing interpretable classification algorithms including decision trees and SHapley Additive exPlanations (SHAP), to provide insights into the distinct characteristics of ASD subtypes, was conducted by Garbulowski et al. [27]. The dataset used is the Autism Brain Imaging Data Exchange (ABIDE) dataset, which includes brain imaging and behavioural data. Quantified results from the study indicated significant dissimilarities between ASD subtypes. The decision tree method achieved a good accuracy in classifying the subtypes, while SHAP values provided interpretations of feature importance, highlighting differences in brain-connectivity patterns and behavioural traits across subtypes.

Diagnosing ASD is a complex process due to its varied symptoms and the subjective nature of current methods like the Autism Diagnostic Interview-Revised (ADI-R), which includes detailed interview responses of ASD-related behaviours. To deal with this issue, Andrade et al. [28] studied diagnosing ASD by combining ML techniques, specifically decision trees and SVMs, with Verbal Decision Analysis (VDA) to create a diagnostic protocol. The approach achieved a diagnostic accuracy of 92%, enhancing diagnostic predictions. More advanced data that consist of video-based contrastive learning has been explored by Ruan et al. [29] using decision trees to enhance diagnostic accuracy. This approach combined video-based contrastive learning with decision trees to analyse and classify ASD-related behaviours, with contrastive learning distinguishing between behavioural patterns by learning from video data. Utilizing video datasets of behavioural actions relevant to ASD diagnosis, the derived models by the decision tree algorithm achieved a high classification accuracy.

Addressing challenges of early ASD diagnosis using classification methods with a focus on rule-based models to facilitate early intervention have been studied by Alwidian et al. [30] and Asgarnezhad et al. [31]. Asgarnezhad et al. [31] focused on techniques like Decision Stump, decision tree, Gradient Boosted Trees, ID3, and CHAID, among others, to develop an ASD model from clinical and behavioural data of individuals with a diagnosis of ASD, achieving an accuracy rate of over 90%. On the other hand, Alwidian et al. [30] evaluated decision trees, Random Forest, SVM, k-NN, and Naïve Bayes on the toddlers autism dataset. The results indicated that models with rules generated by the Random Forest classifier achieved the highest accuracy at 97.2%, outperforming other classification methods like SVM and k-NN. Further, a study that explored the ABIDE dataset, by employing an Enhanced Random Forest (ERF) method, was performed by Qureshi et al. [32]. The results revealed that the ERF method outperforms traditional classification methods by achieving an accuracy of 92%, a sensitivity of 89%, and a specificity of 94%.

Early autism detection in toddlers through behavioural indicators was studied by Chan et al. [7]. The authors utilized various classification techniques and focused on decision trees. The methods employed include decision trees, SVMs, and neural networks, applied to data related to social interactions, communication patterns, and repetitive behaviours. The dataset used in this study includes observational data from clinical assessments of toddlers aged 18–36 months. The results showed that the neural network model achieved the highest accuracy at 94%, followed by the SVM with 91% accuracy, and the decision tree with 87%. A similar study that evaluated a few classification algorithms including AdaBoost, kNN, and ID3, to predict autistic traits in toddlers, was conducted by Rajab et al. [33]. The study used data comprising behavioural features such as communication, social interaction, and repetitive behaviours. The results showed that the AdaBoost algorithm demonstrated the highest accuracy at 95.52%, followed by kNN at 93.15%, and ID3 at 92.10%.

### 3.3. Class Association Rules Studies

Class association rule (CAR) approach combines the strengths of classification- and association-rule mining approaches to enhance predictive accuracy [30]. CARs are rules that associate a specific set of attribute values with a class label, providing a model for classification tasks. Unlike traditional classification methods that may focus solely on prediction accuracy, CARs offer a dual benefit: they not only predict the class of an instance but also reveal useful relationships between attributes and class labels [33]. Incorporating both the frequency and the predictive power of attribute combinations, CARs are particularly useful in research applications like ASD diagnosis where understanding the underlying patterns are as crucial as the predictions themselves.

A predictive Apriori—an association rule mining—was employed to accurately diagnose ASD and extract meaningful patterns from ASD-related data by Sundari [34]. The generated results in terms of the accuracy rate of ASD diagnosis achieved by the Predictive-Apriori algorithm was high in correctly identifying ASD cases. Further, a Constraint Governed Association Rule Mining (CGARM) algorithm, which sets specific constraints to derive rules in order to highlight key genetic markers, was applied by Rajashekar [35]. The authors tried to address identifying significant single nucleotide polymorphisms (SNPs) crucial for ASD classification. Due to the genetic complexity and heterogeneity of autism, revealing key SNPs is difficult. The authors used a dataset consisting of SNPs related to autism, which was processed by the CGARM method and identified strong SNPs, achieving a high classification accuracy.

Alwidian et al. [30] conducted an experimental study on the use of Associative Classification algorithms for predicting ASD. They focused on the Weighted Classification Based on Association Rules (WCBA) algorithm, comparing its performance in terms of accuracy and F-measure against other algorithms on a dataset of 704 instances with 21 attributes. The study found that the WCBA achieved high predictive results. The authors fine-tuned the minimal support and confidence parameters for the Associative Classification algorithms applied to an adult autism dataset. Their results indicated that the WCBA performed well, demonstrating its potential for effective ASD prediction. The comparison included various Associative Classification algorithms such as MCAR, FCBA, ECBA, WCBA, FACA, CMAR, and CBA, with WCBA emerging as the most effective.

### 3.4. Fuzzy Rules

Fuzzy rules are a fundamental component of fuzzy logic systems, which extend classical logic to handle the uncertainty in many applications [36]. Unlike binary logic, where features are either true or false, fuzzy logic permits features to have degrees of truth, ranging between 0 and 1. This flexibility makes fuzzy rules particularly effective for modelling complex, vague, and ambiguous systems. Similar to rules in decision trees or CARS, fuzzy rules are expressed in the form of “if-then” formats, where the antecedent and consequent are defined by fuzzy sets. Widely used in various practical applications such as health informatics, pattern recognition, and decision-making processes, fuzzy rules provide a robust framework for handling uncertainty.

Fuzzy logic systems and a Genetic Algorithm were used to classify ASD severity by Saberipour et al. [37]. The dataset used by the researchers comprised 112 children and adolescents aged 3 to 14, collected from various rehabilitation centers in Tehran. The proposed method achieved a high performance in terms of predictive accuracy. Another study, which employed a deep Multi-Output Takagi–Sugeno–Kang (MO-TSK) fuzzy inference system combined with deep-learning techniques for composite feature learning and ASD classification, was conducted by Lu et al. [38]. The dataset used in this research is ABIDE, and results showed that the proposed method achieves a high classification accuracy, significantly outperforming existing methods, thereby demonstrating its effectiveness in distinguishing between different ASD subtypes.

Hu et al. [39] applied Takagi–Sugeno–Kang (TSK) fuzzy systems on the ASD classification of integrated data from multiple centers with varying characteristics and noise levels. The method proposed is based on TSK fuzzy systems, which model uncertainty and variability in the data effectively to improve the generalizability of ASD classification across different centers. The results showed improvement in classification accuracy, besides enhancement robustness to noise and variability across centers. Moreover, different optimization techniques for fuzzy logic-based ASD diagnosis on the ADI-R dataset were compared by Wanti & Puspitasari [40]. Methods like Genetic Algorithm (GA), Particle Swarm Optimization (PSO), and Simulated Annealing (SA) were employed to optimize the fuzzy logic-model parameters. The optimized fuzzy logic method revealed its potential use as a tool in ASD diagnosis by delivering good results. For instance, accuracy obtained by the fuzzy system exceeded 85% and achieved a sensitivity of 80% and a specificity of 90%.

Table 1 shows the summary of the recent research conducted using rule-based algorithms on the ASD-detection problem.

### 3.5. Hybrid Models

Combining deep learning with feature selection and rule-based classifiers can enhance classifier interpretability, exploration, and accuracy in autism detection. Feature selection techniques enhance efficiency by focusing on the most relevant data, while rule-based classifiers provide explanations for classifier decisions. This integration is particularly valuable in clinical applications, where understanding model behavior is crucial. In this section, we show recent research on integrating deep learning with feature selection and in some cases with rule-based classification algorithms to achieve models’ exploration and interpretability.

Interpretable models are essential in the prediction of autism in domains, where understanding model behavior can lead to better clinical insights and trustworthiness. Combining deep learning with feature selection and rule-based classifiers has emerged as a powerful approach for achieving these goals. For instance, Munroe et al. [44] reviewed applications of interpretable deep learning in neuroimaging, emphasizing the need to balance model complexity and interpretability. The authors highlighted how feature selection techniques, integrated with deep learning, focus on brain regions most relevant to specific conditions, reducing dimensionality while improving model clarity. For instance, results with fMRI data showed that interpretable methods like attention mechanisms enhanced the exploration of brain activity patterns linked to autism, making results clinically actionable and improving model accuracy by 12% when paired with rule-based explanations.

Moreover, Alshammari et al. [4] developed an explainable federated learning framework for autism prediction, integrating deep learning with feature selection to enhance privacy and interpretability. The approach prioritized key features from EEG data while maintaining data privacy across decentralized nodes. By using classifiers with rules to show feature importance, the authors achieved a high accuracy (90%) and better interpretability, demonstrating how specific EEG patterns correlate with autism diagnoses. Similarly, Omrani et al. [45] studies explainable AI for recognizing the faces of autistic children. The authors employed feature selection to identify critical facial attributes before applying deep-learning models, improving interpretability. Rule-based explanations linked these features to behavioural traits, enabling clinicians to understand the model’s predictions, with results demonstrating an increase in accuracy when incorporating rule-based reasoning alongside improved transparency.

Furthermore, Saponaro et al. [46] proposed a deep learning-based joint fusion approach that integrated anatomical and functional brain data to detect autism. Feature selection methods used identified the relevant fMRI and anatomical features, while the learning algorithms validated the contribution of each feature. The hybrid approach improved classification accuracy compared to standalone deep-learning models, highlighting the interactions between feature selection and rule-based classifiers. Another study by Radhakrishnan et al. [47] demonstrated the effectiveness of combining deep learning and Mu-rhythm EEG feature selection in classifying Autism Spectrum Disorder. Their hybrid model achieved medically accepted accuracy.

A study by Prasad et al. [48] employed a deep-learning approach with feature selection to classify ASD using behavioural and imaging data. Feature selection methods used by the authors reduced dimensionality by 30%, while classifiers provided insights into the behavioural traits most relevant to autism. This combination enhanced interpretability and improved model accuracy. Another hybrid approach by Awaji et al. [49] for early ASD detection based on facial feature analysis showed an integration of a CNN algorithm with feature selection to enhance the detection rate. On the other hand, Chen et al. [5] proposed an ASD-detection approach using coarse- and fine-grained facial behavior analysis. The authors used deep-learning models to extract facial features, while feature selection focused on behaviors such as gaze patterns and expressions. This combination enabled precise identification of the significant facial behaviors, improving both interpretability and exploration.

An Attention-Based Hybrid Residual Memory Network (AHRML) for autism detection with feature selection to prioritize relevant fMRI features has been developed by Al- [50]. The authors processed data by attention-based deep-learning techniques and used reasoning algorithm to model interpretability by linking selected features to brain regions associated with autism. This hybrid approach achieved a 94% accuracy rate. On the other hand, Shao et al. [51] integrated deep learning with feature selection to enhance ASD detection using fMRI data. The derived model achieved an 89% accuracy rate, showing the effectiveness of this integrated approach. Fruther, Ismail et al. [52] developed HEC-ASD, a hybrid ensemble-based classification model for predicting autism-related genes. This model used feature selection to identify critical genetic markers and rule-based classifiers for interpretability. The hybrid approach reduced the feature set by 40%, improving classification accuracy.

Recently, Alshammari et al. [4] used rule-based classifiers to explain the relevance of selected EEG features in autism prediction, making the model’s predictions understandable to clinicians. Similarly, Omrani et al. [45] utilized rule-based classifiers to correlate facial features with behavioural traits, enabling actionable insights. In addition, Radhakrishnan et al. [47] showed how rule-based reasoning validates deep-learning outputs by linking EEG features to neurological patterns. This approach improves accuracy and ensures that the model’s predictions align with established clinical knowledge.

Table 2 shows the summary of the recent research conducted using hybrid algorithms on ASD detection problem.

## 4. Discussion

Based on Section 3, the considered research studies have utilized different types of data—primarily behavioural, genetic, and imaging—to classify ASD and employed different rule-based classification techniques. The focus was more on behavioural data [13,33,34] due to the following reasons [17,53,54]:

1. Diagnostic Criteria: The primary diagnostic measures for ASD, as outlined in the Diagnostic and Statistical Manual of Mental Disorders (DSM-5) [55], are based on behavioural traits. These cover communication and social interaction deficits, along with restricted, repetitive patterns of behaviour, interests, or activities.
2. Early Identification: Behavioural data allow for the early detection of ASD symptoms, which is critical for timely intervention. Early behavioural indicators, such as lack of eye contact, limited social engagement, and repetitive behaviours, can be observed in young children, facilitating early screening.
3. Understanding of Traits: Behavioural assessments provide a complete understanding of how ASD displays in daily life. This includes interactions in social settings, responses to sensory requests, and the presence of repetitive behaviours.
4. Validation and Reliability: There are validated behavioural assessment tools that are already established, such as the ADOS and the ADI-R [17,56].
5. Accessibility: Collecting behavioural data is often more accessible and less invasive than genetic or neuroimaging methods. Behavioural assessments can be conducted through observations, interviews, and standardized tests without requiring medical procedures.

While genetic and neuroimaging data provide valued insights into the biological and neurological underpinnings of ASD, behavioural data remain dominant due to its direct relevance to the diagnostic criteria, its role in early detection, and its practicality in various clinical and nonclinical settings. Integrating behavioural data with genetic and imaging data [42,43] may further enhance the performance of the ML algorithms and comprehensiveness of the ASD classification models. Overall behavioural data offer observable and quantifiable traits, genetic data provide insights into the biological basis of autism, and imaging data reveal the neural correlates of autistic behaviours.

There have been many rule-based algorithms used in the considered studies in this paper including CARs such as WCBA, decision trees like Random Forest, C4.5, ID3, etc., rule induction such as RIPPER, and fuzzy rules. The performance of these algorithms when compared with conventional ML techniques varies, and it depends on the input data used, features subsets, algorithms’ hyperparameter settings, and any preprocessing operation, among others. Thereby, it is difficult to compare between these classification algorithms using performance measurements like predictive accuracy, error rate, sensitivity, specificity, F-Measure, etc., unless the same dataset with cases and controls are used. These rule-based methods like CAR, Covering, and induction of rules provided good ASD detection rates by effectively uncovering hidden patterns that are critical for early diagnosis. The use of induction and Covering algorithms allows for easy interpretation and application in clinical settings, where understanding the rationale behind each diagnosis is decisive.

In contrast, researchers like Chan et al. [7] and Ruan et al. [29] used decision tree algorithms such as C4.5 and its predecessor ID3 to model behavioural indicators, benefiting from the robustness and interpretability decision tree learning mechanism, i.e., information gain with Shannon Entropy to reduce uncertainty. For example, Ruan et al. [29] enhanced predictive performance by incorporating more complex data-related video into decision trees, demonstrating flexibility, although potentially at the cost of reduced interpretability compared to pure rule-based models. In addition, Saberipour et al. [36] and Lu et al. [43] utilized fuzzy logic methods, which are adept at handling uncertainty and providing detailed classifications based on symptom severity. Saberipour’s model offered nuanced classifications using fuzzy methods, which is valuable for personalized treatment planning, while Lu’s deep multi-output Takagi–Sugeno–Kang fuzzy inference system integrated behavioural and neuroimaging data, achieving robust performance. However, these models can be more complex and less straightforward to interpret than rule-based classifiers such as CARs, rule induction, and decision trees.

Overall, rule-based classification algorithms in the studies that have been critically analyzed in this research work have shown significant improvement in autism-detection applications since they generate models with high interpretability and rules richness, which offer clear, human-comprehensible insights. These qualities are beneficial in clinical settings, aligning well with regulatory frameworks such as the General Data Protection Regulation (GDPR) [57]. The GDPR emphasizes the “right for elaboration.”, in which individuals’ data processed by automated decisions have the right to understand how those decisions were made. This requirement is clearly fulfilled by rule-based classifiers, as they offer easy-to-explain rules for decision-making. This means that healthcare professionals can provide detailed explanations of the diagnostic process based on specific rules derived from the participants’ data.

The interpretability of rule-based algorithms when compared to more complex machine-learning models, like neural networks, is crucial for clinicians. In autism applications, rule-based algorithms generate decision rules that clinicians can manage and use. For example, a rule induction or decision tree algorithm might identify a sequence of symptoms, such as limited eye contact and repetitive behaviors, leading to a high-risk autism diagnosis. This kind of information is important because it allows clinicians to explain the diagnostic process to patients and their family members, enhancing trust and confidence in the results. The richness of the rules captures large associations within the data. In the context of autism, this richness is particularly useful for identifying ASD indicators, such as subtle social interaction cues or particular non-verbal behaviors. By capturing these detailed patterns, rule-based classifiers can provide more comprehensive diagnostic insights that align well with clinical diagnostic criteria.

While rule-based classifiers offer significant advantages in the classification of ASD as demonstrated in Section 3 and Section 4, they may also face challenges. One limitation in systems generated by fuzzy methods is their reliance on predefined rules, which can lead to reduced flexibility and adaptability in handling the complex nature of ASD symptoms [58]. These classifiers may struggle to capture the full spectrum of behavioural variations and comorbid conditions often associated with ASD, leading to potential misclassifications. Additionally, the process of creating accurate rule sets requires extensive domain knowledge and can be time-consuming.

Another challenge is that rule-based systems often struggle in the ability to learn and improve the models on the fly from new data, limiting their capacity to adapt to evolving diagnostic criteria or newly discovered behavioural patterns. This static nature contrasts with ML models that continuously improve with more data [58]. Furthermore, rule-based classifiers can be sensitive to noisy or incomplete data, which is common in clinical settings, potentially compromising their performance [59]. These challenges highlight the need for integrating rule-based systems with more dynamic ML approaches like deep learning to enhance the robustness and accuracy of ASD classification.

With recent development in AI, it will be advantageous to merge rule-based classification with Explainable AI (XAI) algorithms based on deep learning to offer more realistic framework specially to deal with children with complex ASD activities, gestures, and eye movement. Deep-learning models like Convolutional Neural Networks (CNNs) can identify ASD-related patterns in visual data, but their opacity limits interpretability. Rule-based systems can complement these models by embedding clear, easy-to-understand rules that guide predictions. For instance, combining CNNs with models derived by CARs or rule induction algorithm enables the model to offer high predictions with explainable results that align with clinical expectations.

Recent research highlights the integration of these ML approaches. For instance, Mumenin et al. [60] utilized a deep CNN trained on eye-tracking data to classify ASD and incorporated rule-based explanations to map predictions back to clinically relevant gaze patterns. Similarly, Atlam et al. [61] developed an Explainable Autism Spectrum Disorder Model (EASDM) that integrates domain-specific rules with SHAP, allowing clinicians to understand the rationale behind AI-driven predictions. Lastly, Albahri et al. [62] explored the application of fuzzy logic and multi-criteria decision-making (MCDM) methods to enhance diagnostic prioritization for ASD. The proposed framework integrates explainable AI to address complex health scenarios, improving decision transparency and accuracy. The addition of XAI techniques, such as SHAP or Local Interpretable Model-Agnostic Explanations (LIME), further clarifies model predictions by highlighting the contributions of individual features, enhancing trust and applicability.

ASD is typically diagnosed using standardized medical methods, such as the Autism Diagnostic Observation Schedule (ADOS) and the Autism Diagnostic Interview-Revised (ADI-R) [17,63]. The ADOS involves structured observations of an individual’s social communication skills and repetitive behaviors, whereas the ADI-R collects detailed developmental history through parent or caregiver interviews. These methods assess autism traits and are considered the main ASD criteria in clinical diagnostics. However, these approaches are time-intensive, reliant on subjective evaluation, and require trained medical professionals. Recently, cutting-edge AI technology have been integrated into computer diagnostic tools, such as AI-based systems, analyzing facial expressions, gaze patterns, and movement behaviors. These intelligent tools complement traditional methods by providing consistency, scalability, and early detection capabilities, addressing some limitations.

The integration of rule-based classifiers and advanced AI techniques like deep learning with diagnostic tools in clinical settings can provide an innovative framework for ASD diagnostic tools, offering better decisions and enhancing accuracy, transparency, and alignment with established clinical practices. Deep learning identifies complex patterns in high-dimensional data, and rule-based classifiers complement this by translating the results into understandable diagnostic criteria. Such tools can expand traditional diagnostics like the ADOS and ADI-R by providing objective analysis that reduces the reliance on subjective evaluations.

Deep-learning models analyze large datasets, such as behavioural video recordings or EEG patterns, while rule-based classifiers refine these findings, linking specific features to established diagnostic frameworks, thereby enhancing understandability and clinicians’ exploration of the models. For instance, EEG data showing irregular Mu rhythm patterns can be associated with ASD traits through explicit rules derived from clinical knowledge. These explanations make AI systems more accessible to clinicians and increase their trust in AI-driven tools. This hybrid approach not only improves diagnostic accuracy but also reduces the time and expertise required for evaluation.

## 5. Conclusions

The ASD diagnosis problem involves accurately identifying ASD through clinical assessments and behavioural observations, often complicated by the disorder’s diverse and complex symptoms. Early detection is advantageous as it allows for timely interventions, improving developmental outcomes, enhancing social skills, and reducing long-term care costs, thereby significantly benefiting individuals and their families. Rule-based classification algorithms are a branch of ML approaches that generate “if-then” rules to classify behaviours indicative of ASD, making them crucial in decision-making for clinicians who need to understand and trust the diagnostic process. However, there are limited reviews on the utilisation of rule-based classification algorithms on the problem of ASD diagnosis from a data-science perspective. To fill this gap, this research reviewed and critically analyzed rule-based algorithms for ASD detection. The new review can synthesize current knowledge, highlight strengths and limitations, and identify gaps in existing research related to ASD classification using rule-based classifiers. This review can guide future studies toward more effective and interpretable diagnostic tools, facilitating early and accurate ASD detection. Additionally, it can help clinicians and researchers understand the practical applications and challenges of rule-based models, promoting the integration of these algorithms into clinical practice. By consolidating diverse research findings, the review can also encourage the development of more robust, hybrid models with XAI that deal with complex data and can improve diagnostic accuracy and patient outcomes.

Rule-based classifiers deal with autism classification from a data-driven perspective by offering easy-to-understand explanations for the features identified by the machine-learning models. For example, in ASD detection through facial recognition, a learning algorithm like deep-learning models can extract features such as facial expressions, gaze direction, or microexpressions. Rule-based classifiers then assign importance to these features, explaining how specific traits, such as limited eye contact, relate to ASD characteristics. This approach ensures that the model is not only accurate but also interpretable, enabling clinicians to understand and trust its predictions. Thereby, integrating rule-based classifiers with more emergent learning technologies like deep-learning methods can create advanced AI diagnostic tools for ASD detection by combining high accuracy with explainable models. Deep-learning models, such as convolutional and recurrent neural networks, identify complex patterns in data like fMRI scans, EEG signals, and facial behavior. These methods detect ASD traits that traditional approaches often miss. However, their “black-box” nature makes it challenging to understand the decision-making process, which can limit trust and clinical application. Thus, when rule-based classifiers are used, this drawback can be resolved, making the solution not only effective and efficient but innovative. This is since rule-based classifiers refine this output by establishing clear, understandable links between features and diagnostic outcomes. For instance, they can identify which brain-activity patterns or facial features are most indicative of ASD and explain how these were prioritized during the decision process. The hybrid integration of rule-based classifiers with emergent AI methods can result in ASD diagnostic tools that may offer powerful pattern recognition alongside the transparency required for real-world clinical decision-making.

One of the limitations of this review paper is that it did not look into feature selection and their use within advanced AI techniques like deep learning. Further, the study did not look into the details of deep-learning algorithms, their behaviours, and their upsides and downsides when it comes to ASD detection from complex data like movements or facial expressions. These limitations will be addressed in a separate research study that only focuses on features extraction within deep-learning algorithms for ASD classification.

The use of rule-based classification algorithms for ASD classification offers noteworthy implications for clinical practice. By providing easy-to-explore models by clinicians, rule-based algorithms translate complex data patterns from AI models into explainable if-then rules, bridging the gap between computational tools and human decision-making. Additionally, rule-based algorithms improve diagnostic scalability. Unlike subjective observations, models by rule-based classifiers ensure reproducibility by applying the same diagnostic criteria across cases and controls. This is particularly valuable in resource-limited settings, where access to trained medical professionals may be constrained.

## Figures and Tables

**Table 1:** This shows a summary of the relevant works conducted on ASD using rule-based classifiers.

Year Published	Dataset	Algorithms	Results	References
2020	ASDTests datasets	RIPPER, RIDOR, Nnge, Bagging, CART, C4.5, and PRISM	95% accuracy in RML	[13]
2023	ABIDE dataset	Enhanced Random Forest (ERF)	ERF achieved an accuracy of 92%.	[41]
2021	ASDTests dataset-Toddlers	ID3, AdaBoost, and kNN	The AdaBoost algorithm demonstrated the highest accuracy at 95.52%, followed by kNN at 93.15% and ID3 at 92.10%.	[33]
2021	ABIDE dataset	Decision tree	The decision tree method achieved an accuracy of 85% in classifying the subtypes.	[42]
2023	ASDTests dataset	Fuzzy rules	The proposed fuzzy method achieved an average performance accuracy of 97.4%.	[36]
2022	ABIDE dataset	Hybrid: Deep Multi-Output Takagi–Sugeno–Kang Fuzzy Inference Systems (DMO-TSK FIS)	Quantified results show that the proposed method achieves high classification accuracy, significantly outperforming existing methods, with an accuracy rate of approximately 89.5%.	[43]
2022	ASDTests dataset	Hybrid: Genetic Algorithm (GA), Particle Swarm Optimization (PSO), and Simulated Annealing (SA)	Achieving a diagnostic accuracy exceeding 85%, a sensitivity of 80%, and a specificity of 90%, these outcomes affirm its efficacy in accurately identifying both ASD-positive and ASD-negative cases, emphasizing its importance in enhancing diagnostic precision within ASD assessments.	[40]
2020	ASDTests dataset	Decision trees, Random Forest, SVM, k-NN, and Naïve Bayes	The results indicate that the Random Forest classifier achieved the highest accuracy at 97.2%.	[30]
2020	SNP data that contains genetic markers	CARs and Constraint Governed Association Rule Mining (CGARM)	The CGARM approach successfully identified strong SNPs, resulting in a classification accuracy of 85%.	[35]
2022	QCHAT dataset of toddlers—ASDTests data	Decision trees, SVM, and ANN	The results showed that the neural network model achieved the highest accuracy at 94%, followed by the SVM with 91% accuracy and the decision tree with 87%.	[7]
2021	ADI-R dataset	Decision trees and SVM (SVM), combined with Verbal Decision Analysis (VDA)	The integrated approach achieved a diagnostic accuracy of 92%.	[28]
2023	CalTech interview video database	Decision trees	The integrated approach achieved a classification accuracy of 90%.	[29]
2023	ASDTests dataset	Decision tree algorithms besides conventional ML algorithms like SVMs, Random Forest, and Logistic Regression	The ML model achieved a high accuracy rate of 90% in diagnosing ASD.	[31]
2023	ASDTests dataset related to QCHAT medical questionnaire	Decision tree and the RIPPER rule learner	The rule-based classifier achieved an accuracy rate of 85% in detecting autism in children.	[20]
2020	ASDTests datasets	Decision tree, C4.5, and RIPPER	The ML learning model achieved an accuracy rate of 88% in detecting autism.	[13]
2023	Sample images from the Radboud Faces Database	(WISC-R)	The proposed rule model achieved significant results, with a classification accuracy of 89.5%, a precision of 88.7%, a recall of 90.2%, and an F1-score of 89.4%. These metrics indicate the model’s effectiveness in identifying and generalizing repetition-based behaviours in individuals with autism.	[23]
2020	ASDTests-Child dataset	CARs based on Apriori algorithm	The Apriori algorithm achieved 85% in correctly identifying ASD cases.	[34]

**Table 2:** This shows a summary of the relevant works conducted on ASD using hybrid classifiers.

Year	Dataset	Classification Algorithms	Results	References
2024	Federated EEG Data	Deep Learning, Rule-Based Classifiers, Local Interpretable Model-Agnostic Explanations (LIME)	Achieved 90% accuracy using federated learning for autism prediction. Prioritized key EEG features with rule-based explanations to ensure privacy and interpretability.	[4]
2024	Autistic Children Facial Image Dataset	Deep Learning, Feature Selection, Local Interpretable Model-Agnostic Explanations (LIME), Randomized Input Sampling for Explanation of black-box models (RISE)	Improved accuracy by 15% using interpretable models combining feature selection and rule-based explanations to link facial features to behavioural traits.	[45]
2022	Simons Foundation Autism Research Initiative (SFARI)	Hybrid ensemble-based classification model (HEC-ASD)	Reduced feature set by 40%, achieving a 13% higher classification accuracy for autism-related genes.	[52]
2024	fMRI and Anatomical Data, ABIDE-1	Joint Fusion Deep Learning	Achieved an 18% higher accuracy by integrating fMRI and anatomical data, with feature selection and rule-based validation.	[46]
2024	Mu Rhythm EEG Data	Hybrid Model with Rule-Based Classifiers, Non-linear features	Achieved 92% accuracy, utilizing EEG feature selection and rule-based reasoning for enhanced interpretability.	[47]
2022	Behavioural and Imaging Data	Deep Learning with Feature Selection	Improved accuracy by 10% using reduced-dimensionality data and rule-based classifiers for interpretability.	[48]
2023	Facial Feature Image Data, ABIDE-1	CNN with Hybrid Techniques	Detection rate improved to 88%, integrating CNN-based features with rule-based analysis for early autism detection.	[49]
2024	fMRI Data	Attention-based hybrid optimized residual memory network (AHRML)	Achieved 94% accuracy by prioritizing fMRI features with attention-based models and rule-based interpretations.	[50]
2021	fMRI Data	Deep Learning	Reduced processing time by 25% with improved accuracy of 89% using feature selection and functional connectivity analysis.	[51]
2024	Facial Behavior Data	Coarse- and Fine-Grained Deep Learning	Achieved 91% accuracy by combining coarse and fine-grained facial behavior analysis with rule-based weighting.	[5]
